# *Paracoccidioides*-host Interaction: An Overview on Recent Advances in the Paracoccidioidomycosis

**DOI:** 10.3389/fmicb.2015.01319

**Published:** 2015-11-25

**Authors:** Haroldo C. de Oliveira, Patrícia A. Assato, Caroline M. Marcos, Liliana Scorzoni, Ana C. A. de Paula E Silva, Julhiany De Fátima Da Silva, Junya de Lacorte Singulani, Kaila M. Alarcon, Ana M. Fusco-Almeida, Maria J. S. Mendes-Giannini

**Affiliations:** Faculdade de Ciências Farmacêuticas, UNESP – Universidade Estadual Paulista, Campus Araraquara, Departamento de Análises Clínicas, Laboratório de Micologia ClínicaSão Paulo, Brazil

**Keywords:** *Paracoccidioides brasiliensis*, Paracoccidioides lutzii, fungi–host interaction, paracoccidioidomycosis, *Paracoccidioides* pathogenicity and virulence

## Abstract

*Paracoccidioides brasiliensis* and *P. lutzii* are etiologic agents of paracoccidioidomycosis (PCM), an important endemic mycosis in Latin America. During its evolution, these fungi have developed characteristics and mechanisms that allow their growth in adverse conditions within their host through which they efficiently cause disease. This process is multi-factorial and involves host–pathogen interactions (adaptation, adhesion, and invasion), as well as fungal virulence and host immune response. In this review, we demonstrated the glycoproteins and polysaccharides network, which composes the cell wall of *Paracoccidioides* spp. These are important for the change of conidia or mycelial (26°C) to parasitic yeast (37°C). The morphological switch, a mechanism for the pathogen to adapt and thrive inside the host, is obligatory for the establishment of the infection and seems to be related to pathogenicity. For these fungi, one of the most important steps during the interaction with the host is the adhesion. Cell surface proteins called adhesins, responsible for the first contact with host cells, contribute to host colonization and invasion by mediating this process. These fungi also present the capacity to form biofilm and through which they may evade the host’s immune system. During infection, *Paracoccidioides* spp. can interact with different host cell types and has the ability to modulate the host’s adaptive and/or innate immune response. In addition, it participates and interferes in the coagulation system and phenomena like cytoskeletal rearrangement and apoptosis. In recent years, *Paracoccidioides* spp. have had their endemic areas expanding in correlation with the expansion of agriculture. In response, several studies were developed to understand the infection using *in vitro* and *in vivo* systems, including alternative non-mammal models. Moreover, new advances were made in treating these infections using both well-established and new antifungal agents. These included natural and/or derivate synthetic substances as well as vaccines, peptides, and anti-adhesins sera. Because of all the advances in the PCM study, this review has the objective to summarize all of the recent discoveries on *Paracoccidioides*-host interaction, with particular emphasis on fungi surface proteins (molecules that play a fundamental role in the adhesion and/or dissemination of the fungi to host-cells), as well as advances in the treatment of PCM with new and well-established antifungal agents and approaches.

## Introduction

It is estimated that about 1.2 billion people worldwide suffer from fungal diseases. Some of these are invasive or chronic and difficult to diagnose and treat. It is estimated that 1.5 to 2.000.000 people die of fungal infections each year, surpassing those who die from other causes (Denning and Bromley, 2015). In Latin America, the rich diversity of biomass and climates provides a rich range of habitats for different microorganisms, including these pathogenic fungi responsible for endemic mycoses and that have an important impact on public health: histoplasmosis, coccidioidomycosis, and paracoccidioidomycosis (PCM; Colombo et al., 2011).

The *Paracoccidioides* spp. specie complex is dimorphic fungi that are the etiologic agents of PCM. This is the most important systemic mycosis in Latin America with Brazil, Venezuela, and Argentina being the countries with the greatest number of patients. Non-autochthonous cases have been described outside endemic areas in patients who have lived in or visited Latin America. Brazil, which accounts for 80% of the cases, concentrated the occurrence in its southeastern, southern, and Midwestern regions. PCM is an endemic mycosis that is responsible by the highest cause of mortality and the eighth most important cause of mortality from chronic infectious diseases reaching rates of 1.65 deaths per 106 inhabitants (Coutinho et al., 2002; Bocca et al., 2013). According to Martinez (2010), an estimated 3,360 new cases per year reflect the fatality and mortality rates attributed to PCM in Brazil. Through epidemiological surveys it’s known that PCM occurs throughout Brazil (Blotta et al., 1999; Bellissimo-Rodrigues et al., 2011; Loth et al., 2011; de Souza et al., 2014; Vieira et al., 2014). Recent eco-epidemiological studies (**Table 1**) have been demonstrating the occurrence of the PCM in different regions of the Brazil, warning the scientific community about the importance of this disease to the country.

**Table 1:** Occurrence of paracoccidioidomycosis in the Brazilian territory raised by eco-epidemiological studies.

State	Region	Period	Number of cases	Gender/age	Reference
Parana	Western	2008–2009	102	72 male and 30 female/18–81 years	Loth et al., 2011
Sao Paulo	Southeast	1960–1999	1.000	858 male and 142 female/03–80 years	Bellissimo-Rodrigues et al., 2011
Amazon	North	1997–2012	2.163	1.951 males and 211 females/03–81 years	Vieira et al., 2014
Sao Paulo	Southeast	1988–1996	584	492 males and 92 females/05–87 years	Blotta et al., 1999
Rio Grande do Sul	South	1966–2009	123	104 males and 17 females/02–97 years	de Souza et al., 2014

The *Paracoccidioides* genus is composed of thermally dimorphic fungi classified in the *Onygenales* order and in the *Ajellomycetaceae* family (Untereiner et al., 2004). Currently, due to advances in phylogenetic studies of different *Paracoccidiodes* isolates, this genus is divided into two species: *P. brasiliensis* and *P. lutzii*, being the first divided into three different phylogenetic species, S1, PS2, and PS3 (Matute et al., 2006; Carrero et al., 2008; Marini et al., 2010). Agents of systemic mycoses, such as *P. brasiliensis* and *P. lutzii*, express factors that facilitate their survival in severe conditions inside the host cells and tissues, and as such, benefit the disease’s development (Casadevall and Pirofski, 1999). The successful colonization of host tissues by the fungus is thus a complex event, usually involving various regulatory mechanisms of cellular homeostasis and the expression of different virulence factors during infection that allows the fungi to cause this systemic mycosis with deep extension in the host organism.

The PCM fungi primarily infects male peasants, between 30 and 60 years of age (Svidzinski et al., 1999; Villa et al., 2000), that are mostly represented by rural workers in the endemic areas (Franco, 1987). Poor hygiene, malnutrition, smoking, and alcohol consumption are considered risk factors for the manifestation of the disease (Silva-Vergara et al., 2000). The inhalation of the fungus conidia or mycelial propagules is the most common transmission mode that allows the fungi to reach the lungs (which are the primary infection site).

The clinical forms of PCM were classified based on the relationship between clinical aspects and the natural history of the disease. Then, the infection is related to the patient without signals and symptoms of the disease but with a positive paracoccidioidin skin test reaction. Patients with PCM disease were divided between acute/subacute form (juvenile type), that mainly affects children and young adults presenting more disseminated lesions, and chronic form (that primarily affect adult men) generally found in oral mucosa, airways, and lung lesions (Bocca et al., 2013; Marques, 2013; Martinez, 2015).

Because of its importance, this review will summarize all the recent discoveries on *Paracoccidioides*-host interaction with particular emphasis in fungi surface proteins, which play a fundamental role in the adhesion and/or dissemination of the fungi to host-cells. The goal is to point out that there are multi-factorial processes involving host–pathogen interactions as well as fungal virulence and host immune response. Finally, this review will also focus in the recent advances in drug discoveries and treatments of PCM.

## The Adhesion Process in PCM

The interaction of host and parasite is a complex event in which the host is under pressure to develop resistance while the parasite tries to evade and adapt to the host’s immune response and thus survive in the host’s environment (Tronchin et al., 2008; Sironi et al., 2015).

Paracoccidioidomycosis can be acquired through inhalation of infectious propagules, which then lodge in the alveoli from which they spread to other organs. The fungi developed mechanisms (such as adhesion to host cells), to avoid entrapment within mucus and their elimination by mucigen cilliary cells (Filler and Sheppard, 2006; Hernández et al., 2010). Therefore, their effective adherence contributes to higher speed invasion of host cells, allowing for evasion of the immune system, establishment of the infection, and in the case of *Paracoccidioides* spp., lead to different clinical manifestations (Singer-Vermes et al., 1994; de Oliveira et al., 2015). Another important fact is that *P. brasiliensis* is able to form biofilm *in vitro*, which opens up new possibilities in understanding the infection process of these fungi, since biofilm formation is a condition that provides for the pathogen’s protection against drugs and the host’s immune system (Sardi et al., 2015).

Some differences in the degree of adherence have been observed for *Paracoccidioides* spp. regarding the manner in which they enter different cell types. This is perhaps related to changes in the cell wall composition (Telles-Filho, 1987; Puccia et al., 2011). Hanna et al. (2000) observed differences in adhesion capacity to Vero cells of four *P. brasiliensis* strains. Additionally, successive subcultures of *P. brasiliensis* resulted in their attenuation or loss of virulence (Brummer et al., 1990), that can be re-established after passage in animals (San-Blas et al., 1977; Castaneda et al., 1987) or epithelial culture cells (Andreotti et al., 2005).

Recently de Oliveira et al. (2015) found significantly higher capacity for adhesion to pneumocytes by *P. brasiliensis* compared to *P. lutzii*. They also demonstrated that *P. brasiliensis* is more virulent than *P. lutzii*, using an *in vivo* model. This supports the fact that adherence and virulence are closely related in *Paracoccidioides* spp. and reinforces the importance of adhesion in the infection process of these fungi.

*Paracoccidioides* spp. recognizes several of the host molecules such as components of the extracellular matrix (ECM). ECM is basically composed by collagen, elastin fibers, glycosaminoglycans (GAGS), proteoglycans (PG), fibronectin, laminin, heparan sulfate, nidogen/entactin, hyaluronate, chondroitin sulfate, and collagens subtypes I, III, IV, and V (Dunsmore and Rannels, 1996; Mendes-Giannini et al., 2006; Balestrini and Niklason, 2015). These can serve as a substrate for the attachment of colonizing microorganisms (Chagnot et al., 2012). Comparing the adhesion to ECM components of two species of *Paracoccidioides*, de Oliveira et al. (2015) demonstrated that *P. brasiliensis* adheres more to fibronectin in contrast to *P. lutzii* that showed more tropism to type I and IV collagen.

Different studies reinforced the importance of the fungus interactions with ECM proteins during the adhesion process. González et al. (2008b) evaluated *Paracoccidioides* conidia treated with laminin, fibronectin, and fibrinogen in mice experimental PCM. They observed that the treatment with all ECM proteins, especially laminin and fibrinogen, induces a less severe pathology, with a decrease of chitin content in the lungs. In a different study, André et al. (2004) treated yeasts of *Paracoccidioides* with laminin and they to observed that this treatment induces a less severe pathology.

Several studies in the *Paracoccidioides* genus have been conducted to characterize the adhesion process, revealing that *Paracoccidioides* spp. synthetizes several molecules, known as surface adhesins, that are involved, directly or indirectly, in the interaction with host cells and in the *in vitro* biofilm formation (Mendes-Giannini et al., 2000; Hernández et al., 2010; Sardi et al., 2015).

*Paracoccidioides* spp. adhesins are widely studied using *in vitro* and *in vivo* approaches of different forms of *Paracoccidioides* spp. in order to identify and characterize these molecules. The understanding of the adhesion process provides a better understanding of the disease as well-bringing new possible targets for therapeutics (Gonzalez et al., 2005; Tomazett et al., 2005; Pereira et al., 2007; de Oliveira et al., 2015).

Several *Paracocccidiodes* spp. adhesins have been found to have a multifunctional role, being primarly involved in metabolic pathways and later found in fungus cell walls and/or secreted and mediating fungus adhesion (Karkowska-Kuleta and Kozik, 2014). The transport of these molecules (together with antigenic components and other molecules that can interact with the host’s cellular immune system such as α-galactosyl), to the fungal cell wall and to the extracellular compartment, can be mediated by vesicles produced by the fungus as described by Vallejo et al. (2011, 2012). Recently, Peres da Silva et al. (2015) described the presence of RNA in extracellular vesicles secreted by *Paracoccidioides* spp., which might interfere in both fungi and host gene expression, modulating the host–pathogen interaction.

The gp43 is a glycoprotein that is the most studied molecule from *Paracoccidioides* spp. Due to its importance in the host–pathogen interactions, including the adhesion process. The gp43 was the first described *P. brasiliensis* adhesin, and it binds to laminin and fibronectin. *In vitro* studies showed that treatment with gp43-purified protein is able to reduce *Paracoccidioides* adhesion, showing that this protein is one of the mediators of fungus adhesion to host epithelial cells and internalization (Vicentini et al., 1994; Hanna et al., 2000). This interaction also occurs with macrophages cells. Silenced strains for gp43 are less adherent or internalized by activated macrophages (Almeida et al., 1998; Torres et al., 2013).

A 32 kDa hydrolase (PbHAD32) was found in *Paracoccidioides* spp. cell wall extracts from mycelial and yeast forms and is able to bind to laminin, fibronectin, and fibrinogen and act as an adhesin in the initial stage of *Paracoccidioides* spp. adhesion. An increase of PbHAD32 is observed during the transition from conidia to yeast or mycelial form (González et al., 2005, 2008a; Hernández et al., 2012). Hernández et al. (2010) using antisense RNA (aRNA) technology performed PbHAD32 silencing and the knockdown of this gene resulted in morphological changes in the yeast form. Furthermore, a decrease of the adherence of both yeast and conidia forms to epithelial lung cells (A549) in a knockdown strain (*PbHAD32 aRNA)* was observed. *In vivo* analysis demonstrated a significant increase in the survival rate of mice challenged with *Paracoccidioides PbHAD32 aRNA* when compared to the wild type *Paracoccidioides* (Hernández et al., 2010, 2012).

The 30 kDa protein was first identified in a *Paracoccidioides* proteomic study before and after mice infection, where a significant increase of its expression was observed after mice infection and was also characterized as a laminin ligand (Andreotti et al., 2005). Later, da Silva et al. (2013) sequenced this protein and identified it as belonging to the 14-3-3 protein family characterized as small multifunctional proteins present in eukaryotic cells. During infection, Pb14-3-3 accumulates in the *P. brasiliensis* cell walls. The treatment with recombinant protein, promotes the inhibition of *P. brasiliensis* adhesion to epithelial lung cells, demonstrating its importance in this process (da Silva et al., 2013).

In a study conducted by Donofrio et al. (2009) to identify fibronectin-binding adhesins from *P. brasiliensis*, a 54 kDa protein was highly expressed in different strains when cultured in a medium supplemented with blood. The sequencing of this protein identified it as enolase (PbEno), a well-known glycolytic enzyme. Later, it was found that surface expressed PbEno also binds to laminin, type I and IV collagen, plasminogen, and fibrinogen (Nogueira et al., 2010; Marcos et al., 2012). The participation in the adhesion process was evaluated using anti-54 kDa polyclonal antibodies in which the inhibition of *P. brasiliensis* adhesion to epithelial lung cells *in vitro* was demonstrated (Nogueira et al., 2010). In addition, during infection and when cultivated with sheep blood, an increase of PbEno in cell walls was observed, demonstrating its role in fungus–host interaction (Marcos et al., 2012). The ability of enolase to interact with plasminogen has already been related to the pathogen’s invasive capacity. It is mediated by lysine-dependent binding, degrading ECM, and promoting invasion (Ghosh and Jacobs-Lorena, 2011). Sequence analysis showed that PbEno has similar internal motif responsible for plasminogen binding in *Paracoccidioides* spp. and the recombinant PbEno binding to plasminogen in the presence of a lysine, suggesting that PbEno also plays a role in the invasion process (Marcos et al., 2012).

Triosephosphate isomerase (TPI) is a glycolytic enzyme described as a protein that is able to react with the sera of PCM patients. The localization of TPI was detected in the cytoplasm and in the cell wall of the yeast phase of *P. brasiliensis* (da Fonseca et al., 2001). Pereira et al. (2007) produced an anti-TPI polyclonal antibody and used it to treat *P. brasiliensis* and observe the influence of this treatment in the interaction of the fungi with epithelial cells. After the treatment, they observed that the antibody inhibited the adhesion of *P. brasiliensis*. These findings showed the involvement of the TPI in the cell adhesion acting as an adhesin.

Malate synthase is an enzyme from the glyoxylate pathway and in *Paracoccidioides* spp. is also required in allantoin degradation pathway (Zambuzzi-Carvalho et al., 2009). However, besides the metabolic role, PbMLS is found in fungus cell walls and is characterized as an adhesin able to bind to fibronectin as well as types I and IV collagen (da Silva Neto et al., 2009). In a study of intermolecular interactions of PbMLS, de Oliveira et al. (2013) found that PbMLS present in cell walls interact with other adhesins such as enolase and TPI and this interaction could enhance the adhesion ability.

GAPDH (glyceraldehyde 3-phosphate dehydrogenase) is a well-known protein from the glycolysis pathway, however, this protein can also act as virulence factor for some pathogens, including fungal pathogens (Karkowska-Kuleta and Kozik, 2014; Marcos et al., 2014). In *P. brasiliensis*, GAPDH expression is increased during the mycelium-yeast transition and was found in cell wall and extracellular vesicles (Barbosa et al., 2004; Longo et al., 2014). *Paracoccidioides* spp. GAPDH is able to bind to host ECM components laminin, fibronectin, and type I collagen. This molecule seems to play a role in the initial steps of infection once *in vitro* assays demonstrated the inhibition of adhesion and infection of *P. lutzii* to pneumocytes after fungus incubation with anti-GAPDH antibody or cell treatment with recombinant GAPDH (Barbosa et al., 2006).

Another protein involved in the process of cell adhesion and tissue invasion/dissemination is the fructose 1,6 bisphosphate aldolase (ALD) protein that interacts with plasminogen. The antibodies anti-ALD and the recombinant protein were able to reduce the interaction between macrophages and *Paracoccidioides* (Chaves et al., 2015).

Recently, de Oliveira et al. (2015) evaluated gene expression of differents adhesins as 14-3-3, ENO, gp43, MLS, GAPDH, and TPI after mice infection by *P. brasiliensis* and *P. lutzii*. They could observed that 14-3-3 and enolase were the most expressed adhesins and also that *P. brasiliensis* express in higher levels adhesins than *P. lutzii*. Besides this, this study demonstrated that the virulence of its species is related to its adhesion capacity with *P. brasiliensis* being more virulent than *P. lutzii*.

**FIGURE 1:**
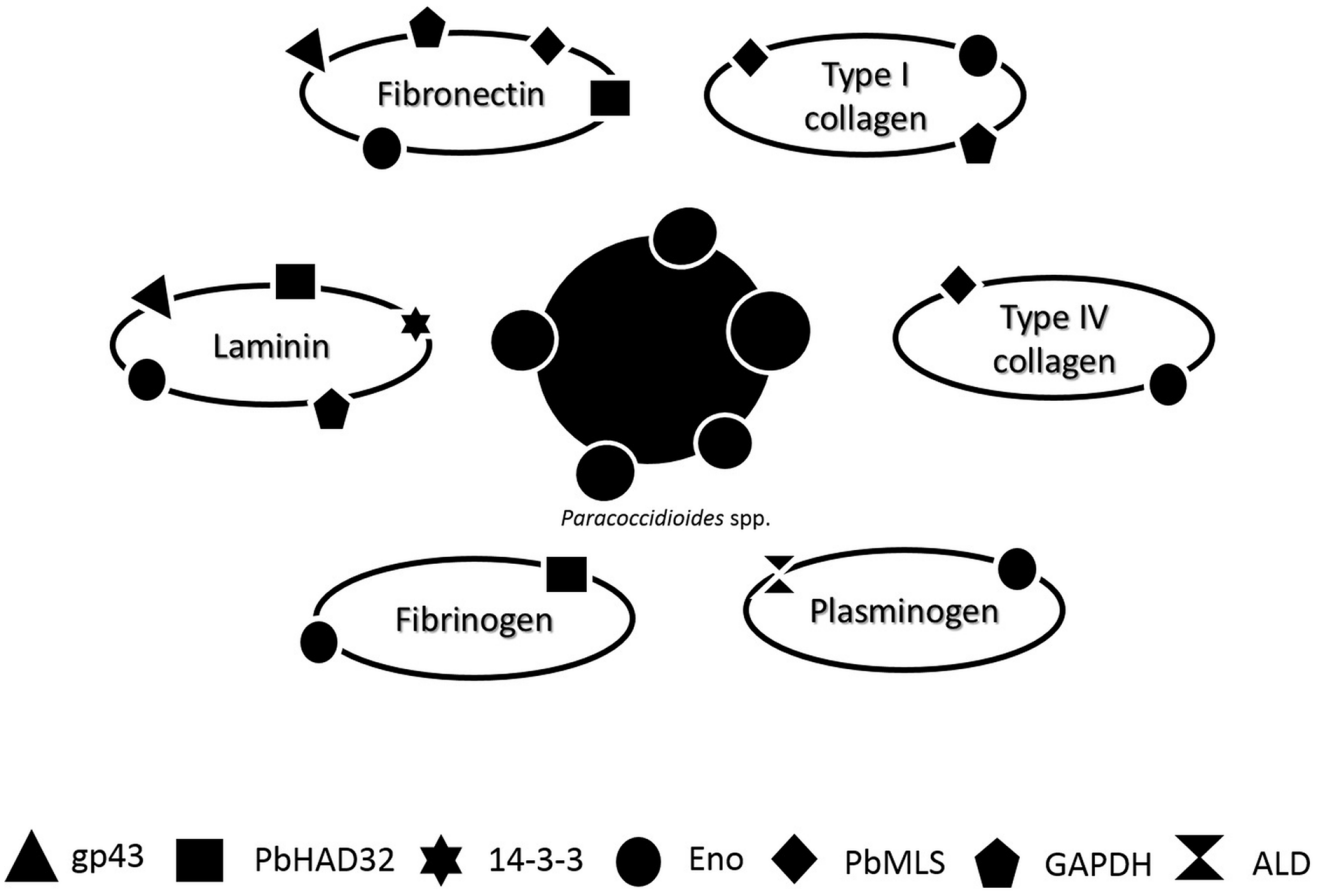
**Schematic representation of the affinity of different *Paracoccidioides* spp. adhesin [gp43, hydrolase (PbHAD32), 14-3-3, Enolase (ENO), Malate synthase (MLT), glyceraldehyde 3-phosphate dehydrogenase (GAPDH), and 1,6 bisphosphate aldolase (ALD)] to different ECM components: laminin, fibronectin, type I collagen, type IV collagen, plasminogen, and fibrinogen**.

The adhesion is an universal prerequisite for pathogens to efficiently deploy their repertoire of virulence (Krachler and Orth, 2013). In summary, the attachment of *Paracoccidioides* spp. to host cells is mediated by adhesins present at the fungal surface and that this is a critical step in PCM, acting as an essential virulence factor for *Paracoccidioides* spp. **Figure 1** summarizes the affinity of each adhesin to different ECM components: laminin, fibronectin, type I collagen, type IV collagen, plasminogen, and fibrinogen.

Based on all that was presented in this section, what is needed are new approaches that aim for the discovery of new molecules or further investigation of the already known molecules. This holds especially true for studies that evaluate strategies to block the adhesion in order to try and discover how the fungi modulated itself to cause the infection and how we can avoid the infection and prevent the PCM.

## Morphological Switching and Pathogenicity of *Paracoccidioides* spp.

Different polysaccharides, proteins, lipids, and melanin compose the complicated structure of fungi cell walls. An incisive way for pathogens to respond, adapt, and survive in new niches of infection can be found in the alterations in the expression of surface-exposed molecules under different environmental conditions (Karkowska-Kuleta and Kozik, 2015).

The genus *Paracoccidioides* comprises of thermally dimorphic fungi that grow as saprophytic mycelium at environmental temperature (e.g., 26°C; San-Blas, 1993). Produces infective conidia or mycelial (M) fragment propagules that are inhaled by the host. When the propagules reach the pulmonary alveolar epithelium of a mammalian host (exposed to temperature higher than 37°C), they transform into the parasitic yeast (Y) form causing different clinical manifestations. This phenomenon can also be reversibly triggered *in vitro* by changing the temperature from 26 to 37°C (San-Blas et al., 2002).

The glycoproteins and polysaccharides network that composes the cell wall of *Paracoccidioides* spp., similar to many other fungi, is important for the protection against environmental stresses (De Groot et al., 2005) while promoting its virulence. The morphological switch, a mechanism for allowing the pathogen to adapt and thrive inside the host (Nemecek et al., 2006), is obligatory for the establishment of the infection. This seems to be related to pathogenicity, since isolates incapable of undergoing the morphological transition are less virulent (Maresca and Kobayashi, 2000).

The fungal cell wall synthesis is performed by glucan synthases (Sorais et al., 2010). The phenotypic switch entails changes in the composition of the fungal cell walls with a predominance of β-1,3 and β-1,6-glucan and carbohydrates in mycelial form, while in yeast form there is a prevalence of α-1,3-glucan and chitin (Puccia et al., 2011; Free, 2013). Changes may occur in the quantity and the spatial arrangement of these polysaccharides (San-Blas et al., 1994). This occurs in order to ensure fungal survival within the host (Tavares et al., 2015). α-1,3-glucan is correlated with the level of virulence (Hogan and Klein, 1994), hiding immunostimulatory β-glucans that could be detected by the host phagocytic cells (Klein and Tebbets, 2007), assisting its evasion of the host’s immune responses (Rappleye et al., 2007).

For *Paracoccidioides* spp. the temperature is an essential factor involved in dimorphism and is preceded by several molecular changes (Boyce and Andrianopoulos, 2015). This is a characteristic shared with other dimorphic fungi such as *Histoplasma capsulatum*, *Blastomyces dermatitidis* (Medoff et al., 1987), *Coccidioides immitis, Sporothrix schenckii*, and *Penicillium marneffei* (Klein and Tebbets, 2007). The growth of yeast from the mycelium is initiated from the time of thermal change reaching 50% of changes after 48 h from the start of the process. The multiple developments into yeast budding occurs after 5 days (Salazar and Restrepo, 1985; Goldman et al., 2003). The reverse has also been reported and studies show that 48 h following the temperature change, 50–60% of the cells showed conversion mycelium (Goldman et al., 2003; Nunes et al., 2005).

However, in *Paracoccidioides* genus the conidia- or mycelium-to-yeast transition is blocked by exposure of *Paracoccidioides* spp. to female hormones, such as estrogen, via steroid-binding proteins (Loose et al., 1983; Aristizábal et al., 1998). This gives the PCM the peculiarity of affecting more men compared to the number of women (Shankar et al., 2011b).

Regarding the evaluation of molecular mechanisms and gene expression in both morphological states, several studies have been conducted using different strategies such as expressed sequence tags (ESTs) libraries, microarrays, analysis of genes expressed during the stages of mycelium and yeast, as well as those differentially expressed in transition and proteomics. Several efforts have been made to understand the morphological alterations, including those depending on the factors of temperature and the presence of female hormones. There are many studies focusing on genes that are regulated during mycelium-to-yeast (M-Y) transition (Felipe et al., 2005; Bastos et al., 2007; Parente et al., 2008; Muñoz et al., 2014). Although a number of genes that govern the phase transition are known, how these genes fit into a larger network of regulated genes remains poorly explored (Gauthier, 2015).

Transcriptional analysis of 6,022 assembled groups demonstrated that the mycelial cells have a more aerobic metabolism in comparison to the yeast phase, with greater expression of genes of citrate cycle such as succinyl-CoA dehydrogenase and isocitrate synthase suggesting a metabolic shift to oxidative phosphorylation. In contrast the yeast phase displays slanted energy metabolism for the production of alcohol by fermentation, presenting the glyoxylate pathway (into anaerobic metabolism) as being more active. This is demonstrated by analysis of transcription in yeast and mycelia which is consistent with low oxygen levels found in infected tissue (Felipe et al., 2005).

A biochip carrying of 4,692 genes from *P. brasiliensis* was used to trace gene expression in different points of the morphological transition (5–120 h). Among the various genes identified, some encoding enzymes are involved in the catabolism of amino acids, signal transduction, protein synthesis, cell wall metabolism, genome structure, response to oxidative stress, growth control and development (Nunes et al., 2005).

Proteomic analysis during phase conversion of *P. brasiliensis* demonstrated quantitative differences correlated with transcripts levels. The mycelia phase protein profile showed 18 overexpressed proteins involved in cell defense, energy, and protein fate. During M-Y transition, 33 proteins were upregulated, most of them belonging to the glycolytic pathway. Some glycolytic enzymes such as enolase and fosfoglucomutase begin to accumulate during the transition (M-L) and maintain high levels in the yeast phase. It is therefore another sign of the global reorganization of carbohydrate metabolism that occurs during morphological change (Rezende et al., 2011).

N-*linker* glycans are involved in glycoprotein folding, intracellular transport, secretion, and protection from proteolytic degradation (Nagai et al., 1997). In *Candida albicans* it has been shown to be involved in cell wall integrity as well as in the fungus–host interaction (Mora-Montes et al., 2007). Dos Reis Almeida et al. (2014) showed that the treatment of *Paracoccidioides* with tunicamycin, responsible for blocking the N-*linked* glycosylation of α-1,4 amylase, interfere in the transition for both Y-M and M-Y, since the α-1,4 amylase is responsible for biosynthesis of α-1,3 glucan the major cell wall glucan of the yeast form.

Phosphatidic acid and diacylglycerol produced by a phospholipase D1 participates in the morphological transition of *C. albicans* (Hube et al., 2001). A similar finding was seen in *P. brasiliensis* in which up-regulation of phospholipase was found in M-Y transition (Soares et al., 2013).

Thermal dimorphism may occur as a result of a specialized heat shock response triggering a cellular adaptation to high temperatures (Lambowitz et al., 1983). Matos et al. (2013) demonstrated the involvement of HSP90 during the dimorphism of *P. brasiliensis* using pharmacological approaches. HSP90 is required for the transition from non-infective to infective forms but not for Y-M transition. This protein is also highly transcribed under *in vitro* oxidative stress. HSP90 is a chaperone that binds and stabilizes calcineurin. It also competes with calmodulin for the Ca^2+^/calmodulin docking site in calcineurin interfering with the activation of the latter (Imai and Yahara, 2000). So, it was suggested that HSP90 acts synergistically with calcineurin in the control of cell differentiation (Matos et al., 2013). Other proteins of HSP family such as HSP70, HSP80, and HSP88, were down-regulated in mycelial cells treated with estradiol (Shankar et al., 2011b) suggesting that this hormone impairs the favorable expression of genes necessary for adaptation to a change of temperature (Nicola et al., 2008).

In spite of several studies suggesting the potential role of estradiol in dimorphism of *Paracoccidioides*, the exact mechanism that leads to such genetic modulation resulting in differences in disease rates, remains unknown (Tavares et al., 2015). Hormones act as messenger molecules, leading to regulation of gene expression through receptor-mediated interactions that mediate this interaction and the subsequent functional response to the presence of the hormone (Shankar et al., 2011a).

*In vivo* studies have shown that female mice, especially at estrus, reach a higher clearance of yeast and restraint of fungal proliferation as compared to male mice (Aristizábal et al., 1998, 2002; Sano et al., 1999). Pinzan et al. (2010) revealed remarkable influences of gender on experimental PCM, which could be partly attributed to interference of female hormones on the immune response triggered by a *P. brasiliensis* infection. Estradiol promotes protective responses to this infection, IL-12, IFN-γ, and TNF-α cytokines (Calich et al., 1998; Kashino et al., 2000) correlated with resistance to female infection. On the contrary, infected male produces IL-10 (Benard et al., 2001; Oliveira et al., 2002; Romano et al., 2002) which plays an important role in antigen specific immunosuppression of PCM (Pinzan et al., 2010).

Shankar et al. (2011b) using microarray technology to evaluate the *P. lutzii* transcriptional response to a fixed concentration of estradiol during 9 days, revealed that the chitin synthase 1 gene (CHS) was down-regulated in response to estradiol at earlier time points. Nunes et al. (2005) identified a positive modulation of chitin synthases and down-regulation of chitinases in the M–Y transition, while Bastos et al. (2007) detected two chitinases over-expressed in the dimorphic transition. In fact, the yeast cell wall is mainly constituted of chitin (37–48%), compared to the mycelium form (7–18%; Kanetsuna et al., 1969).

High levels of gene expression may occur during this process. Hernández et al. (2011b) showed increased expression of HSP90, AOX, and GS1 (glucan synthase-1) throughout the entire yeast to mycelium germination and αGS (glucan synthase α) for the opposite. The HSP90 was up regulated early in the transition suggesting their involvement in the initial contact of the fungus with the host and the modifications necessary to adapt within the same. The AOX (alternative ubiquinol oxidase) gene acts by reducing the reactive oxygen species and correlates with metabolic activation required to obtain carbon and energy owing to the non-phosphorylative nature of the alternative respiratory pathway to the morphological changes (Gessler et al., 2007; Hernández et al., 2011a). AOX is present in early stages of M-Y transition and plays an important role in intracellular redox balance. Furthermore, it is the only enzyme in *P. brasiliensis* that is not present in its mammalian hosts therefore it is a promising target for therapy (Martins et al., 2011).

TNF-α is a cytokine related with anti-microbicidal activity in IFN-γ activated macrophages stimulating NO production. Gonzalez et al. (2004) illustrated that TNF-α activated macrophages are capable of inhibiting the conidia to-yeast transition in *P. brasiliensis* by an NO-independent pathway; acting as a fungicidal and/or fungistatic mechanism against *P. brasiliensis* conidia.

Cyclic AMP (cAMP) is the regulatory component of a well-characterized signaling pathway implicated in a variety of cellular processes among fungal species (Fernandes et al., 2005). The importance of the cAMP-signaling pathway in the control of morphological changes and pathogenicity of various fungi has been reported (Medoff et al., 1987; Cho et al., 1992; Borges-Walmsley and Walmsley, 2000). During morphological transition the cAMP levels increase transiently in the early stages (<24 h) and progressively in the subsequent stages (>120 h; Chen et al., 2007). Moreover, the transition may be modulated by exogenous cAMP (Paris and Duran, 1985; Chen et al., 2007), suggesting possible involvement of cAMP in the dimorphic transition.

Understanding how different cell types recognize both yeast and mycelial and how each cell type is activated in accordance with the morphology is important, however, the likely consequences of this activation probably differ according to cell type (e.g., in macrophage or in epithelial cell; Jacobsen et al., 2012). Inhibitors targeting the morphological transition from mycelium-to- yeast are an interesting choice to attempt to control the *Paracoccidioides* infection. As discussed above the change to yeast form is essential for the establishment of infection, and thereby inhibitors of this can prevent the infection. **Table 2** summarizes the main works related to dimorphism of *Paracoccidioides* spp. that focused on specific genes and proteins.

**Table 2:** Summary of studies related to dimorphism of *Paracoccidioides* genus.

Condition	Approach (isolate)	Target	Observation	Reference
Mycelium-to-yeast transition	Pharmacologycal tools – inhibition with geldanamycin (*P. brasiliensis)*	Hsp90	Up-regulated	Matos et al., 2013
Mycelium-to-yeast Yeast-to-mycleium	Real-time reverse transcription-polymerase chain reaction	Hsp90/AOX/GS1 αGS	Up-regulated Up-regulated	Hernández et al., 2011b
Mycelium-to-yeast transition	Pharmacologycal tools – inhibition with CsA, a calcineurin inhibitor cyclosporine A (*P. brasiliensis)*	Calcineurin	Transition to the yeast form was blocked before the blastoconidial budding stage	Campos et al., 2008
Mycelium-to-yeast transition	Transcriptional profiling and pharmacological tools – 4-HPPD inhibitor (*P. brasiliensis*)	4-hydroxyl-phenyl pyruvate dioxygenase (4-HPPD)	Up-regulated Inhibit growth and differentiation to the pathogenic yeast phase	Nunes et al., 2005
Mycelium-to-yeast	Pharmacologycal tools – inhibition with benzohydroxamic acid – inhibitor of AOX (*P. brasiliensis)*	AOX	Delayed the M-Y transition	Martins et al., 2011
Yeast-mycelium germination and mycelium/conidia-to-yeast transition	Antisense RNA technology (*P. brasiliensis*)	AOX	Delayed the Y-M transition	Hernández et al., 2015
Mycelium-to-yeast transition	Transcriptional response to 17-β-estradiol treatment (*P. brasiliensis)*	Chitin synthase	Down-regulated in response to estradiol	Nunes et al., 2005
Mycelium-to-yeast transition	1007 ESTs from a transition cDNA library (*P. lutzii*)	Two chitinases	Up-expressed	Bastos et al., 2007
Mycelium-to-yeast transition	Gene expression in the presence or absence of 17-β-estradiol (*P. lutzii*)	Rho-GTPase components FKS1 and AGS (coding α-1,3-glucan synthase)	Down-regulated	Shankar et al., 2011b
Mycelium-to-yeast and yeast-to-mycelia	Pharmacological approach – using tunicamycin (TM)	N-glycosylation	TM treatment interferes the transition in both directions by interference in the activity of α-1,4 amylase (involved in the biosynthesis of α-1,3 glucan)	Dos Reis Almeida et al., 2014
Mycelium-to-yeast	Real-time (*P. brasiliensis*)	Phospholipase (PLB)	Up-regulation in mycelial cells	Soares et al., 2013

## Host Cells Manipulation by *Paracoccidioides* spp.

The ability of pathogens to colonize their hosts is highly dependent on mechanisms that may allow the pathogen to break the physical and immunological barriers imposed by the host. In order to avoid rapid clearance of the organism, pathogens act quickly and effectively on adhesion to host cells.

The capacity of cells to interact with each other in an orderly manner depends on multiple adhesive interactions between cells and their adjacent extracellular environment, mediated by cell adhesion molecules (Miyoshi and Takai, 2008; Troyanovsky, 2009). These function as cell surface receptors that can trigger physical and biochemical signals that regulate a great numbers of functions such as cell proliferation, gene expression, differentiation, apoptosis, and cell migration and are used as a gateway to some pathogens (Finlay and Falkow, 1997; Guttman and Finlay, 2009; Han et al., 2010; Voltan et al., 2013).

Many pathogenic microorganisms have the ability to induce its internalization in epithelial cells, forcing the activation of phagocytosis mechanism. Specific extracellular signals can stimulate their cytoskeleton rearrangement in the contact site with the microorganism, making cells to behave like a “phagocyte unprofessional” (Swanson and Baer, 1995; Lim and Gleeson, 2011), in a process that involves integrins and the cytoskeleton (Mendes-Giannini et al., 2004; Feriotti et al., 2013). *Paracoccidioides* spp. invasion affects the structure of the cytoskeleton of pulmonary epithelial cells and keratinocytes, interfering with morphological aspects of actin, tubulin and cytokeratin (Mendes-Giannini et al., 2004; Peres da Silva et al., 2011).

The capacity of fungal invasion to mammalian cells is specific to some fungi and there is still a lacuna in the understanding of this process (Tsarfaty et al., 2000; Wasylnka and Moore, 2002; Mendes-Giannini et al., 2004). The signaling pathways that control the morphological changes in *P. brasiliensis*, as well as the cellular signals upon interaction with the host cell are also not well-understood. Mendes-Giannini et al. (2004) showed that treatment with cytochalasin D and colchicine reduced the invasion by *P. brasiliensis*, indicating the functional involvement of microfilaments and microtubules in this process.

Some studies evaluated the role of adhesins in the invasion process of *Paracoccidioides* and it was observed that gp43 may also participate in the cytokeratin degradation leading to the loss of the filamentous characteristics that can facilitate the invasion of the host (Mendes-Giannini et al., 1990; Puccia and Travassos, 1991; Tacco et al., 2009; Puccia et al., 2011). The 14-3-3 adhesin is also known to have the capacity to cause structural modifications in the host cells influencing in the polymerization of the cytokeratin microfilaments of actin (Andreotti et al., 2005; Mendes-Giannini et al., 2006; da Silva et al., 2013).

The Rho GTPase family of proteins is known to regulate the dynamic organization of the cytoskeleton and membrane traffic physiological processes such as cell proliferation, motility, polarity, and growth (Sinha and Yang, 2008). The Rho-GTPase is able to down-regulate genes related to chitin and glucans biosynthesis. Rho-GTPase, FKS (β-1,3 glucan synthase), and AGS1 (coding α-1,3 glucan synthase) were down-regulated on *Paracoccidioides* estradiol samples treated. They indicate that this hormone promotes a transcriptional modulation of the cell wall, remodeling related genes (Shankar et al., 2011b; Tavares et al., 2015). Rho GTPases have been extensively studied in human fungal pathogens and have a set of interacting proteins to orchestrate their activation in the cells (Yamochi et al., 1994). Cdc42, a member of the Rho GTPase, was characterized as a convergence point in the signal transduction and are involved in multiple signaling pathways including receptor tyrosine kinases and cytokines, heterodimeric G proteins, as well as physical and chemical stress. In *P. brasiliensis* plays a role in morphology of yeasts cells since the knockdown PbCDC42 showed decrease in cell size and more homogenous cell growth and this provided a higher phagocytosis and decreased virulence (Almeida et al., 2009).

In mammalian cells, the Rho GTPases are also the center of a complex signaling pathway that plays an important role in adhesion. The activation of tyrosine kinase (PTK) receptors stimulate Rho GTPase which in turn activates the Ras pathways and MAPKs (Sinha and Yang, 2008). Monteiro da Silva et al. (2007), showed significant inhibition of fungal invasion after pre-treatment of epithelial cells with genistein, a specific inhibitor of PTK located on the plasma membrane of the epithelial cells. These results suggest that the inhibition of PTK is important in signal transduction during early events in the adhesion and invasion processes of *P. brasiliensis* in epithelial cells.

Apoptosis is a highly regulated physiological process of cell death required for the development and homeostasis of multicellular organisms by eliminating individual cells without inducing an inflammatory response. The enabling or prevention of apoptosis can be a critical step in the development of infectious processes. The process of apoptosis is characterized by typical changes in the symmetry of the plasma membrane, chromatin condensation, nuclear fragmentation, DNA cleavage, cell disintegration, and formation of apoptotic bodies (Strasser et al., 2011).

Programmed cell death has been observed as a response to a variety of infections and can be mediated by a variety of virulence determinants encoded by pathogens. The modulation induced by pathogens pathways responsible for cell death in the host favor the elimination of cells of the immune system or avoidance of host defense response that attempt to act in eliminating the infection (Weinrauch and Zychlinsky, 1999).

The ability of the pathogen to induce apoptosis in phagocytes may be an important virulence factor, since it reduces the host’s defense mechanisms (Ashida et al., 2011). *P. brasiliensis* and other fungi can induce the apoptosis of phagocytes to acquire advantages, allowing intracellular survival in epithelial cells (Cacere et al., 2002).

*Paracoccidiodes brasiliensis* induces apoptosis when it invades epithelial cells or phagocytes, which benefits its intracellular survival (Souto et al., 2003; Mendes-Giannini et al., 2004, 2005; Verícimo et al., 2006; Ketelut-Carneiro et al., 2015). Silva et al. (2008), showed that *P. brasiliensis* induces apoptosis of macrophages by expression of caspase-2, 3 and 8, but also found that it induces the expression of genes that encode inhibitors of apoptosis proteins, such as caspase-8 and Fas-L inhibitors. Caspases-2 and 8 are responsible for the transduction of signals for cleavage of other caspases, such as caspase-3, which leads to apoptosis induction (Silva et al., 2008).

*Paracoccidiodes brasiliensis* may modulate apoptosis of epithelial cells A549 by the expression of apoptotic molecules such as Bcl-2, Bak, and caspase-3, confirming the inducing of apoptosis by the fungus which can then survive and spread to other parts of the body (Del Vecchio et al., 2009). More recently, Silva et al. (2015), showed that the 14-3-3 and gp43 adhesins has strong influence in this process. Campanelli et al. (2003) demonstrated that apoptosis mediated by Fas-FasL and CTLA-4 engagement are involved in modulating the immune response in patients infected with PCM. Souto et al. (2003) demonstrated in experimental PCM, a considerable increase in apoptosis in the infection site. Cacere et al. (2002) studied the role of apoptosis in PCM using peripheral blood mononuclear cells of patients with the PCM disease, noting that apoptosis induced by gp43 was lower in controls than in peripheral blood mononuclear cells of patients.

**FIGURE 2:**
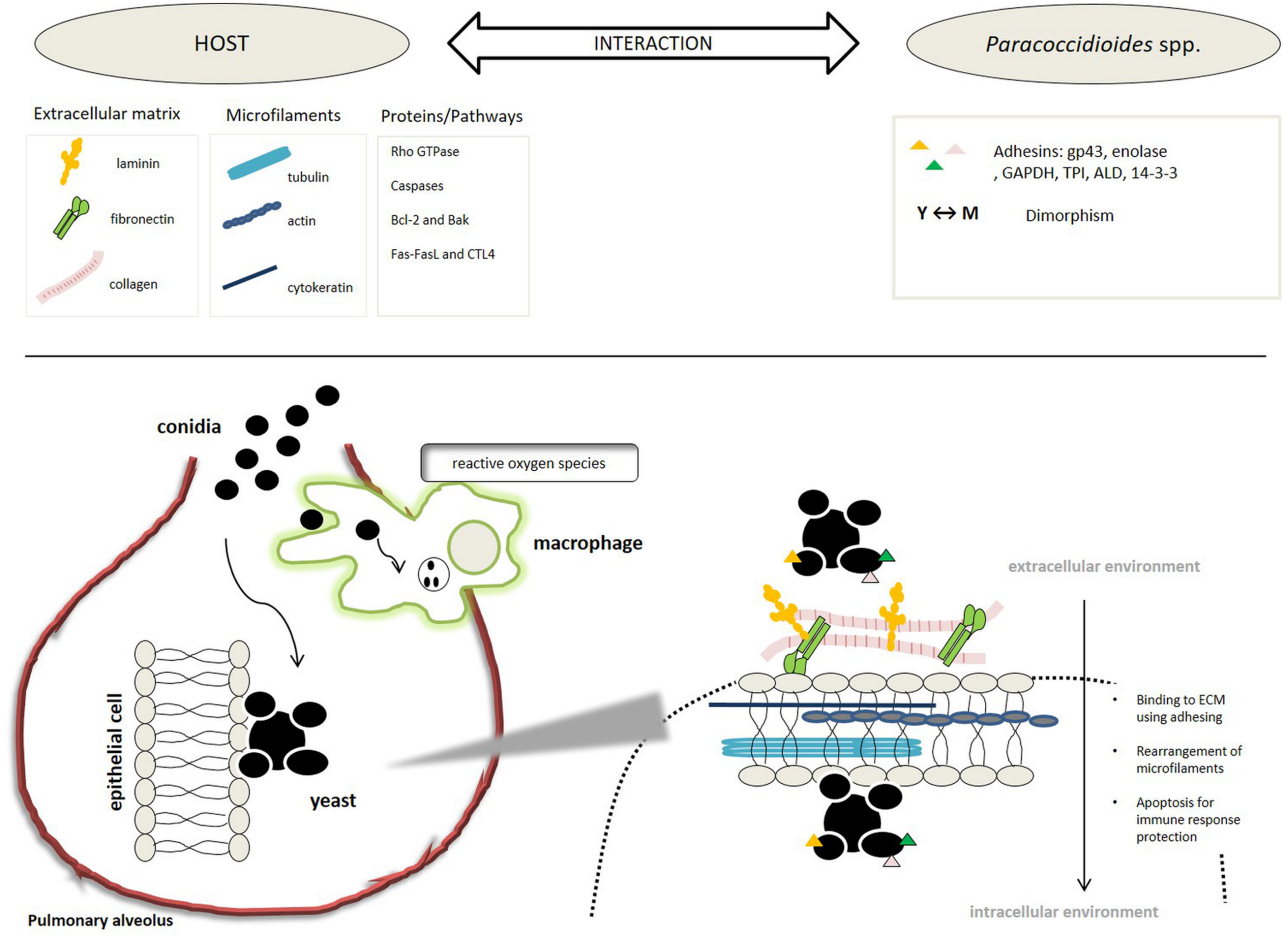
**Schematic representation of the steps, processes, and molecules involved in the *Paracoccidioides*-host interaction**.

All of these studies demonstrated that *Paracoccidioides* spp., during its evolution, has developed mechanisms that allows the fungi, since it is inhaled in its mycelial form, to survive in a hostile host environment. During the interaction, using its adhesins, *Paracoccidioides* spp. adheres to host ECM components and cells, manipulates the cell cytoskeleton, invades the cytoplasmic compartment, and can then induce the cell apoptosis, which gives it its capacity to evade the immune system and spread within the host organism causing systemic mycosis, as summarized in **Figure 2**.

## Advances in Animal Experimentation for the Study of the PCM

The use of animals in research is essential for studies of host–fungal interaction, pathogenesis, treatment mechanisms, immunological aspects, or studies that imply the response of a complex organism. It is important to consider the experimental animal type, age, sex, and routes of inoculation (Conti-Diaz et al., 1959; Iabuki and Montenegro, 1979; Defaveri et al., 1982). The firsts studies described guinea-pigs (de Brito and Netto, 1963), hamsters (Iabuki and Montenegro, 1979), and rabbits (Conti-Diaz et al., 1959) as models to study PCM, however, most of the animals do not develop the disease until a long time after being infected (Linares and Friedman, 1972).

After many decades of investigation, murine animal models are considered the gold standard for *in vivo* studies to simulate the *Paracoccidioides* spp. infection. The establishment of a pulmonary PCM was described in murine models using the intratracheal route after about 30 days of infection. Antibodies were detected 15–60 days after infection, however, were not observed after 360 days (Defaveri et al., 1982). Intranasal route was efficient to develop fatal acute pulmonary or chronic pulmonary and disseminated PCM by using different inoculum concentrations. The development of this model of infection is useful to study treatment (fatal acute pulmonary) and to understand immunological aspects of the disease (chronic; Brummer et al., 1984). The dissemination occurs through a hematogenous route and affects mainly the lungs, secondly liver, thirdly the lymph-nodes and finally within the spleen with the formation of granuloma (Bedoya et al., 1986). The classification of nine different congenital strains of mice were realized after infecting these animals using an intraperitoneally route with *P. brasiliensis*. These mice were classified according to the susceptibility to infection as very resistant, intermediate, and sensitive. The study demonstrated that the susceptibility to *P. brasiliensis* infection of the different animal strains was not dependent on the inoculum concentration. In addition, male mice were generally more susceptible to the infection than females (Calich et al., 1985). In the infection of susceptible mice, high numbers of viable yeasts in different organs were found, however, low fungal burden were observed in all examined organs of resistant animals representing the regression of the infection (Singer-Vermes et al., 1993).

Once the infection model was established to study the efficacy of traditional medicines, new drug candidates or drug combinations could be evaluated (Lefler et al., 1985; McEwen et al., 1985; Restrepo et al., 1992), as well as to study immunologic aspects (Defaveri et al., 1989; Calich and Kashino, 1998; Kashino et al., 2000; Pinto et al., 2006).

Because of the advances in medical technology the number of animals in the research increased and many acts and laws were created in different countries to control ethical issues and to minimize the pain to animals during experimentation (Doke and Dhawale, 2015). Since 1959, the use of animals during scientific experiments has been a debated from which the three Rs theory was created. This theory proposes for the **R**eduction of the number of animals used in an experiment, the **R**efinement of the experiment to animal welfare, and the **R**eplacement of animals by using alternative methodologies (Russell and Burch, 1959).

The use of alternative models like tissues or cell cultures, computer or mathematical analysis (*in silico* testing), and imaging/analyzing techniques are suggested to obtain preliminary data before the start of *in vivo* assays. However, in many cases the research requires information about the response of one whole and complex organism (Balls, 2002; Arora et al., 2011; Doke and Dhawale, 2015).

Considering the need for animal experimentation and the restriction in the use of mammalian animals because of ethical issues, researchers developed alternative animal models. Invertebrates represent a good alternative for *in vivo* assays, because of their short life cycle, small size, evolutionary conservation of the innate immune response between invertebrates and mammals, and low cost. A large number of animals can be used per experiment and until now, no ethical problems limit their use (Mylonakis et al., 2007; Lionakis, 2011; Wilson-Sanders, 2011; Glavis-Bloom et al., 2012; Arvanitis et al., 2013).

Different invertebrate model have been used to study fungal diseases. The fly, *Drosophila melanogaster* (Alarco et al., 2004; Lionakis and Kontoyiannis, 2012), the nematode, *Caenorhabditis elegans* (Pukkila-Worley et al., 2009, 2011; Muhammed et al., 2012); the insect *Galleria mellonella* (Fuchs et al., 2010; Mesa-Arango et al., 2013; Firacative et al., 2014; Maurer et al., 2015) were reported as being used to study fungal virulence factors and to identify novel antifungal compounds.

The use of alternative animal models to study PCM was firstly described by Thomaz et al. (2013). In this study, *G. mellonella* model was used to study *P. lutzii* that was able to kill larvae at 25 and 37°C. Moreover, melanization and granuloma-like structures were observed. Recently, because of the new classification of two distinct species (*P. brasiliensis* and *P. lutzii*), a comparative study of the virulence was developed. Both species cause a hemocyte decrease and kill *G. mellonella* in a similar way. However, *P. lutzii* has higher adhesion ability to hemocytes and this could be attributed to the higher expression of the gp43 gene (Scorzoni et al., 2015). To study the importance of the adhesins for the virulence of *P. brasiliensis* and *P. lutzii*, the treatment of *Paracoccidioides* spp. with antibodies to block two adhesins (14-3-3 and enolase) caused a decrease in the death of the larvae (de Oliveira et al., 2015).

Despite the evolutionary distance between invertebrate and mammalian models, recent works describe the correlation response to infection between these models (Brennan et al., 2002; Brunke et al., 2015; Desalermos et al., 2015). Considering these evidences, invertebrate models are a good alternative for preliminary studies to investigate *Paracoccidioides* spp. virulence, as well as new treatment and immunological aspects of the infection.

## Facing the Problem: Through the Diagnostic to Treatment of PCM

The diagnosis of PCM is based on clinical and laboratory findings. In the acute or juvenile form of the disease, the skin lesions are often present. On the other hand, in the chronic or adult form, the lung is mainly affected. In this case, it is indicated by a radiography of the organ, which exhibits a pattern that resembles a butterfly wing, characterized by bilateral and symmetrical reticulonodular infiltrate in the two upper thirds of the lungs (Barreto et al., 2012). In the laboratory, the direct microscopic examination of the material collected from lesions or tissue is made to observe the fungi, especially its typical multiple budding aspect, as well as the culture to observe the thermal dimorphism, but the fungus has slow growth (Mendes-Giannini and Fusco-Almeida, 2013; Benard and Mendes-Giannini, 2014).

Furthermore, the diagnosis of disease can be made using serological methods. The counter-immunoelectrophoresis (CID) and double immunodiffusion (IDD) are the reactions most used in reference centers (Vidal et al., 2014). In these cases, the 43 kDa glycoprotein (gp43), which is secreted during the infection, is the main antigen detected. The values of titers correlate with the severity of the disease and efficacy of the treatments. In addition, the negativity or stabilization at dilution 1:2 or less indicates the disease cure (Abreu e Silva et al., 2013). However, differences in the antigenic composition, probably related to phylogenetic peculiarities of the two species, should be considered in the diagnosis of PCM (Batista et al., 2010).

When detection by microscopy and serology fail, an alternative can be the use of molecular techniques as polymerase chain reaction (PCR) can be used (due to its greater sensitivity). Several studies have been designed with specific primers to target the genes of the *Paracoccidioides* species. For example, primers for the gp43 antigen were developed to identify *P. brasiliensis* DNA (Gomes et al., 2000). A set of primers for PbITS1s and PbITS3a genes was also used for the detection of the fungus by PCR (Buitrago et al., 2009). Another study reported the use of the primer OPG18, which generates two specific DNA fragments (0.72 and 0.83 kb) for *P. brasiliensis* (San-Blas et al., 2005). Finally, Motoyama et al. (2000) showed that the use of fungal universal primers to target 5.8S and 28S rDNA genes followed by more specific primers (OL3 and UNI-R) for PCR resulted in good identification of *Paracoccidioides* spp.

Recently, Nobrega de Almeida et al. (2015) proposed the use of MALDI TOF MS for *Paracoccidioides* spp. identification. In this study, they analyzed 22 strains, belonging to the two species of the genus. All of the strains were correctly identified. MALDI TOF MS is an interesting tool because of its possibility to adapt to routine laboratories and because the results achieved by this study brings benefits in the clinical and laboratorial studies allowing for the identifying of differences between the diseases caused by this genus.

Besides all well-established methodologies to diagnose the PCM, the diagnostic is not an easy subject. The observation of the fungi in clinical specimens, and growth and reversion to mycelium phase, is difficult in clinical labs. Because of this, until recent times, the serological diagnosis was the most commonly used, since molecular approaches are expensive in countries that PCM occur. However, several recent studies in the characterization of different isolates of *Paracoccidioides* spp. bring difficulties to the serological diagnosis.

Gegembauer et al. (2014) for example, demonstrated that serum from PCM patients infected with *P. brasiliensis* is not able to recognize any antigen from the cell-free preparations of *P. lutzii*, however, serum from patients infected with *P. lutzii* is able to recognize both antigens from *P. lutzii* and *P. brasiliensis*. This means that *P. lutzii* serum is more complex antigenically presenting species-specific antigens and common antigens shared with *P. brasiliensis*. Queiroz Júnior et al. (2014) analyzed the protein/glycoprotein profiles of exoantigens from two clinical isolates of *P. brasiliensis* and three of *P. lutzii* with differences between the species observed. *P. lutzii* exoantigens were different from each other showing high species-specific antigens variability, while *P. brasiliensis* isolates exoantigens present similar protein profiles.

Because of these difficulties in the identification and diagnostic of the PCM with incidence of false negative results (da Silva et al., 2015), this is a public health problem as the number of notifications of the disease can be higher than the numbers we currently have today. In addition, the correct identification of the infection can lead to an efficient treatment. Because of this, new efforts in the identification of serological markers is extremely necessary and one of the great challenge in the study of PCM.

Many drugs are useful in treating PCM. Ribeiro in 1940 suggested the initial treatment with sulfapyridine. Later, Lacaz, and Sampaio proposed the use of amphotericin B in 1958. Barbosa e Vasconcelos, in 1973, recommended the use of a combination of trimethoprim-sulfamethoxazole. Around 1979, Negroni suggested the use of ketoconazole. Restrepo, in 1987 suggested itraconazole and more recently, in 2007, the use of voriconazole was suggested by Queiroz-Telles (Cavalcante et al., 2014).

The treatment depends on the severity of the disease, type of antifungal agent, and the time of use. Despite the limited information on studies with different therapies, the itraconazole therapy is the first choice to control the mild to moderate clinical forms. Since 1987, many groups developed studies with azoles antifungals, which showed a reduction in the symptoms, and that they arrested the progression of the PCM (Negroni et al., 1987a,b,c; Restrepo et al., 1987). However, itraconazole therapy is not easily available in most of the endemic regions. Consequently, the therapy consisting of a trimethoprim-sulfamethoxazole combination (daily for 12 months for mild cases and for 24 months in moderate clinical infections) is a useful option. On the other hand, for severe cases, amphotericin B therapy is the best choice. In case of PCM of the central nervous system, the treatment should be with fluconazole or voriconazole therapy daily for 3–6 months, with a maintenance dose daily for 6–12 months. This is because both have a good penetration through the blood brain barrier (Marques, 2012). Today, ketoconazole is little used for the treatment of this infection because of its severe side effects (hepatotoxicity, loss of libido, inhibition of cortisol production etc; Ferreira, 2009).

The possibility of triazole derivatives interacting with several drugs has to be kept in mind such as antihistamines, antacids, H2 receptor blockers, barbiturates, cyclosporine, diphenylhydantoin, digoxin, cisapride, and rifampicin, among others, as well as the well-known side effects and toxicity (nephrotoxicity, myocardial toxicity, myelotoxicity, etc.) related to amphotericin B which will sometimes require discontinuation of therapy (Ferreira, 2009). In the last 30 years there have been efforts at improving AmB preparation, however, the high costs, neglected clinical data, and alternative antifungal therapies have led to the use of this therapy as a second-line therapy (Laniado-Laborín and Cabrales-Vargas, 2009).

More recently, Rodríguez-Brito et al. (2010) evaluated the susceptibility of *P. brasiliensis* (both at their mycelial and yeast phase), to caspofungin, an antifungal drug of the echinocandin class. For the yeast phase, they found that caspofungin was able to inhibit the growth in 20–65%, while in the mycelial, 75–82%. This variation in their susceptibility is related to the amount of cell wall β-1,3-glucan, that the caspofungin target, which is more pronounced in the mycelial than in the yeast phase of the fungi. These results are interesting and new studies in the use of this drug in the treatment of PCM should be made, especially in studies using combinations of caspofungin with other antifungal drugs to increase their inhibitory capacity.

There are not many reports in the literature about resistant yeasts of *Paracoccidioides* spp. to antifungal therapies. There is a study however, that relates clinical and *in vitro* resistance to ketoconazole and trimethoprim-sulfamethoxazole. In this study, they have found that patients infected with *P. lutzii* had good responses to trimethoprim-sulfamethoxazole, while those infected with *P. brasiliensis* relapsed with the same drug administration (Hahn et al., 2003). In a different way, another study verified that the melanization process decreased susceptibility to antifungal agents, particularly amphotericin B, what can lead to resistance (da Silva et al., 2006).

Due to these facts, new drugs that are safer, more effective, cheaper and with shorter periods of therapy, seem warranted for the treatment of PCM. In this sense, many groups have been developing new alternatives treatment.

One trend is the study of natural and semi-synthetic compounds with great biological activity. In 1989 there began the evaluation of the antifungal activity of Ajoene, a compound derived from ethanolic garlic extracts. These inhibited the growth of *P. brasiliensis* by affecting the integrity of the fungal cytoplasmic membrane (San-Blas et al., 1989). The same authors discussed the possible involvement of ajoene on the sulphydryl metabolism of *P. brasiliensis*, inhibiting the effect on the yeast cells but not on the mycelial cultures (San-Blas et al., 1993). Alterations was observed in phospholipid, fatty acid proportions, phosphatidylcholine, and phosphatidylethanolamine in both phases and reduced saturated fatty acids in the yeast phase, with a corresponding increase in the unsaturated components (San-Blas et al., 1997). Two studies evaluated the antifungal effect of the ajoene in murine models were published; one showing a significant reduction in the levels of antibodies at the 10th week of treatment (Maluf et al., 2008). The other showed a positive additive effect when ajoene therapy was used in association with antifungal drugs (sulfametoxazol/trimethoprim) and a protective proinflammatory immune response (Thomaz et al., 2008).

Martins et al. (2009) showed that *Paracoccidioides* spp. isolates were susceptible to curcumin, a compound produced by the rhizome of *Curcuma long*, and which presented more inhibition effect than the antifungal agent, fluconazole. Johann et al. (2010a) found that the extract from *Schinus terebinthifolius* presented strong antifungal activity against *P. brasiliensis* isolates. Another study from the same authors showed that two compound isolates from the extract of *Schinus terebinthifolius*, schinol, and a new biphenyl compound, had antifungal activity against *P. brasiliensis* isolates. Schinol presented a synergistic effect when combined with Itraconazole (Johann et al., 2010b).

The 6-quinolinyl and quinolinyl N-oxide chalcones, specifically those named 4c and 4e, presented strong activity against *P. brasiliensis.* Histopathological analysis and a progression score of the disease in mice showed that the 4c compound was able to control inflammation and resolved the infection with better results than treatment with Itraconazole and 4e, while avoiding granuloma formation and preservation of lung tissue (de Sá et al., 2015).

Gullo et al. (2012) evaluated natural and semi-synthetics compounds such as maytenin and pristimerin and observed excellent minimum inhibitory concentration against different isolates of *P. brasiliensis*. In the same way, de Paula et al. (2014) evaluated the antifungal activity of the Alkyl gallates, which presented important biological activity reported by the literature, against different fungi species, including different isolates of *P. brasiliensis* and *P. lutzii*. They observed that these molecules presented important relations between the structure and activity, and that the decyl gallate have special activity against *Paracoccidioides* species.

Clinical and experimental data indicate that cell-mediated immunity plays a central role in host defenses against infection by *P. brasiliensis*, whereas high levels of specific antibodies and polyclonal activation of B cells are associated with more severe forms of disease (Cano et al., 1998).

The gp43 contains epitopes capable of producing a cellular immune response in guinea pigs (Rodrigues and Travassos, 1994) and human patients (Saraiva et al., 1996). The sensitivity of the immune response in mice to gp43 occurs by proliferation of CD4 + Th1 (Travassos et al., 1995). These epitopes stimulate CD4 + Th1 lymphocytes, which produce interferon (IFN-γ), which has the function of stimulating the formation of granulomas that may contain yeasts (Brummer et al., 1988). However, the contribution of each subtype of T cell in the immune response of the host depends on the genetic patterns and an immunity with a balance of CD4/CD8 regulating the secretion of cytokines of the Th1 and Th2 type, which correlates with resistance of the host to infection by *P. brasiliensis* (Chiarella et al., 2007).

A 43 kDa glycoprotein (gp43) has 416 amino acids, where a specific stretch of 15 amino acids designated as P10, is recognized by T lymphocytes in mice and humans. The protective effect of P10 is related to inducing an immune response of Th1 dependent IFN-γ- dependent on isogenic mice (Taborda et al., 1998).

Isogenic mouse strains immunized with P10, developed lung infection 200 times less intense than the unimmunized animals (Taborda et al., 1998). Iwai et al. (2003) using TEPITOPE software verified the probability of caucasian HLA-DR recognizing different peptides. They verified that P10, a promiscuous peptide, was an important vaccine candidate for use in humans (Iwai et al., 2003). This peptide could be associated with drugs commonly used for the treatment of PCM and presented an additive effect in the experimental model using BALB/c mice. This demonstrates the capacity of peptide P10 to be useful for reducing the treatment time of this mycosis (Marques et al., 2006). Besides this, Magalhães et al. (2012) demonstrated the potential use of primed dendritic cells (DCs) with P10 as a vaccine that can protect the host against the development of PCM or treating a well-established disease.

According to studies developed in the last years, innate immune system and DCs play an important role in the resolution of *Paracoccidoides* spp. and other dimorphic fungal infections (Thind et al., 2015). DCs play a crucial role in the detection of pathogens, trigger an initial response of the host, as well as instruction to the adaptive immune response. It is also known that DCs play an important role in the induction of effector T cells that *P. brasiliensis* infection control and has been shown that *P. brasiliensis* induces regulatory DCs in susceptible mice. This, in turn, promotes IL-10 production and contributes to the infection susceptibility (Ferreira et al., 2007). Recently, Dos Santos et al. (2011) demonstrated that *P. brasiliensis* infection stimulates migration of DC and, bone marrow-derived DC, when stimulated by *P. brasiliensis*, migrate to the lymph nodes and activate a T-cell response. These studies open up new perspectives since the understanding of the regulation of the DC migration allow for the development of tools to efficiently activated a T-cell response aiding in the control of PCM.

The latest research about alternative therapies presented the immunization in murine models with rPb27, a recombinant protein of *P. brasiliensis*, showing its protective effect against the PCM and its important ability to prevent pulmonary fibrosis (Reis et al., 2008; Fernandes et al., 2011a,b; Morais et al., 2015).

**FIGURE 3:**
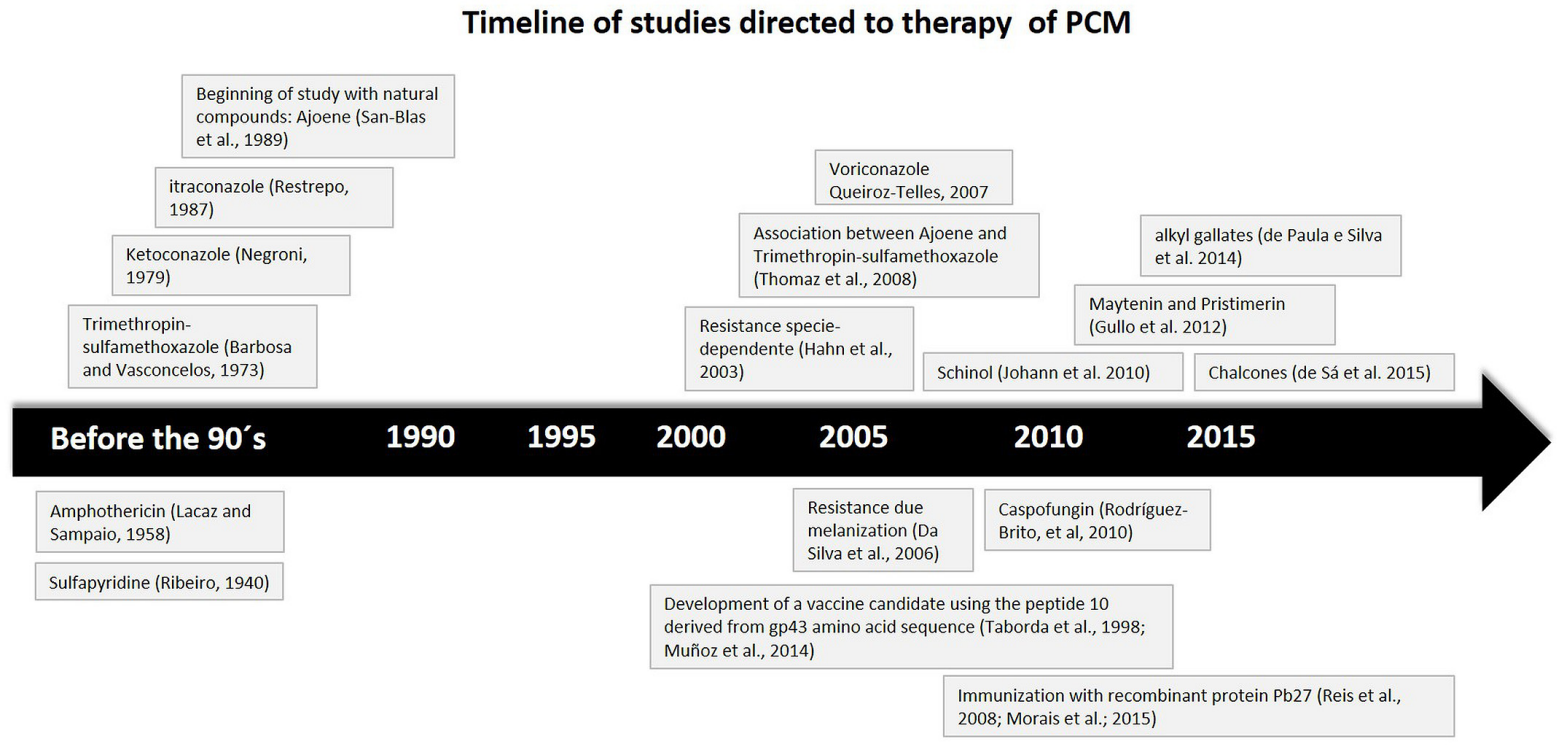
**Time line based on studies that brings development and new insights on treatment and active biomolecules against PCM**.

The **Figure 3** we present a time line based on studies that bring development and new insights into the treatment and use of active biomolecules against PCM.

The treatment of mycosis is a great challenge for science, and in the PCM, this is not different. The problem is even more challenging because since 2006, no antifungal was approved. Of the drugs available today, many cannot be orally administered, have high toxicity, display cases of resistance, and present drug interactions. Thus, the development of new antifungal therapies has become an increasingly challenging problem mainly because of their growing resistance. This issue leads to a search for new antimicrobial agents that have different mechanisms to effectively combat infections and that do not contribute to the resistance of the pathogens that may complicate any therapy (Krachler and Orth, 2013).

## Conclusion Remarks

The advances in PCM studies bring us a better knowledge of how the interaction with the host was constructed during its evolution enabling the fungi to evade from host human immune system and remain in the organisms causing a mycosis with a high incidence in Latin America. This disease is a great public health issue that, with agricultural expansion, has an increasing occurrence area that may affect many more people in the future. This expansion is an alarming problem since the detection of the disease is difficult depending on the isolate, the patient, and the fact that the treatment of the PCM is difficult given the limited arsenal available against it.

The studies we present in this review are evidence of a great effort in the search for knowledge of the PCM and its etiologic agents, *P. brasiliensis* and *P. lutzii*, in the last years. The details of the *Paracoccidioides*-host interaction, the advances in the use of animal models to study the disease, and the discovery of new treatment methods and anti-*Paracoccidioides* agents, reveal a promising future in combating this disease.

## Conflict of Interest Statement

The authors declare that the research was conducted in the absence of any commercial or financial relationships that could be construed as a potential conflict of interest.
